# Novel volumetric imaging biomarkers for assessing disease activity in eyes with PCV

**DOI:** 10.1038/s41598-022-06742-2

**Published:** 2022-02-22

**Authors:** Chinmayi Himanshuroy Vyas, Chui Ming Gemmy Cheung, Janice Marie N. Jordan-Yu, Hitoshi Shimizu, Anna Cheng Sim Tan, Shaun Sebastian Sim, Beau James Fenner, Masahiro Akiba, Usha Chakravarthy, Kelvin Yi Chong Teo

**Affiliations:** 1 Singapore Eye Research Institute, Singapore National Eye Centre, 11 Third Hospital Avenue, Singapore, 168751 Singapore; 2 Duke-NUS Medical School, National University of Singapore, Singapore, Singapore; 3 Topcon, Tokyo, Japan; 4 Ophthalmology and Vision Sciences, Queen’s University, Belfast, UK

**Keywords:** Health care, Medical research

## Abstract

The aim of this study was to evaluate influence of baseline imaging features on visual and anatomical outcomes in eyes with PCV treated with anti-VEGF monotherapy. In this prospective study we enrolled participants with treatment-naïve PCV who followed a treat-and-extend protocol using intravitreal aflibercept (IVA) monotherapy. Baseline clinical features evaluatedincluded best corrected visual acuity (BCVA), traditional features such as lesion size, fluid-related OCT parameters and novel parameters using automated software. This included quantitative and qualitative pigment epithelium detachment (PED) parameters [height, volume]; and choroidal parameters. [choroidal thickness (CT), choroidal volume (CV) and choroidal vascularity index (CVI). We evaluated the predictive value of each parameter on visual and anatomical outcome at month 12. We additionally evaluated initial treatment response after 3 monthly injections with respect to month 12 outcomes. Fifty-two eyes from 52 participants were included in the study. The BCVA increased from 61.1 ± 13.2 to 69.6 ± 13.2 early treatment diabetic retinopathy study (ETDRS) letters (*p* < 0.01) and CRT reduced from 455.7 ± 182.4 µm to 272.7 ± 86.2 (*p* < 0.01) from baseline to month 12. The proportion of eyes with PED decreased significant from 100% at baseline to 80% at month 12 (*p* < 0.01). Reduction in the mean maximum height of PED (from 381.3 ± 236.3 µm to 206.8 vs ± 146.4 µm) and PED volume (from 1322 ± 853 nl to 686 ± 593 nl) (*p* < 0.01) was also noted from baseline to month12. Baseline features associated with better month 12 BCVA included baseline BCVA (β =  − 0.98, 95%CI − 3.38 to − 1.61, *p* = 0.02) and baseline CRT (β =  − 0.98, 95%CI − 1.56 to − 0.40, *p* = 0.04) while the disease activity at month12 was significantly associated with lower baseline CRT (366.0 ± 129.5 vs 612.0 ± 188.0 , *p* < 0.001), lower baseline PED height (242.0 ± 150.0 vs 542.0 ± 298.0 µm, *p* < 0.01), lower baseline PED volume (0.6 ± 0.3 mm^3^ vs 2.2 ± 1.3 mm^3^ vs, *p* < 0.01), lower proportion with marked CVH (17.9% vs 46.2%, *p* = 0.02) and lower mean CVI (61.8 ± 1.4 vs 63.0 ± 1.4, *p* < 0.02). Additionally, a larger decrease in CRT (per 100 nm) and larger PED volume reduction (per 100 nl) at month 3 from baseline were associated with greater BCVA gain and inactive disease. PED-related volumetric parameters have an additional predictive value to traditional biomarkers of disease activity in eyes with PCV undergoing anti-VEGF monotherapy. With increasingly precise quantification, PEDs can be a crucial biomarker in addition to traditional parameters and may aid in retreatment decisions.

## Introduction

Optical coherence tomography (OCT) has provided valuable information regarding the retinal microstructure to guide treatment and prognosticate outcome in eyes with neovascular AMD treated with anti-vascular endothelial growth factor (VEGF)^1–3^. In current clinical practice, non-monthly regimens like treat and extend (TAE) and pro re nata (PRN) relies heavily on disease activity detected on OCT and other modalities to dictate the retreatment criteria. These well-recognized imaging biomarkers include the presence of sub/intra retinal fluid on OCT and haemorrhage detected on clinical examination.

Polypoidal choroidal vasculopathy (PCV) is considered a subtype of neovascular age related maculopathy (nAMD) which is characterized by polypoidal lesions at the termini of a type 1 macular neovascularization detected on indocyanine green angiography (ICGA)^4–8^.

Anti VEGF monotherapy for PCV has been popularized in clinical practice^9,10^. After the favourable visual outcomes demonstrated in clinical trials such as PLANET and ALTAIR^11,12^. These studies also base retreatment decisions on disease activity assessed by a combination of clinical and OCT-based criteria. Specifically, increase in fluid on OCT was considered evidence of disease activity. However, beyond IRF and SRF, two OCT-based imaging features have been proposed to be particularly relevant in eyes with PCV, but are currently not routinely incorporated into clinical assessment^13–22^. Firstly, the influence of pigment epithelial detachment (PED), on visual outcome remains poorly understood^21,23–29^. Secondly, choroidal congestion is also increasingly believed to be key in the pathophysiology and subsequent treatment response but are inconsistently evaluated in prior studies.

Inconsistencies in results from previous studies regarding the influence of PED and choroidal congestion may be due to the lack of standardized quantification methods. Most prior studies use rudimentary, suboptimal quantitative measurements of single point measurement of both PED and sub-foveal choroidal thickness^18,19,30^. These measurements may not capture changes within the larger macula region which a volumetric measurement can. Recent advances in segmentation algorithms coupled with manual correction can help address the shortcomings in the accuracy of PED and choroidal measurements. These algorithms can generate segmentation lines to delineate PED and choroid throughout the macula volume scan, allowing accurate thickness and volumetric measurements of the different retinal layers and the choroid within the central 6 mm of the posterior pole. In addition, there has been increasing interest in evaluating whether choroidal parameters such as choroidal hyperpermeability (CVH), or choroidal vascularity index (CVI) may influence treatment response^14,31–33^.

We recently reported the results of a randomized clinical trial comparing two regimens of intravitreal aflibercept in eyes with PCV^34^. The mean BCVA improvement was + 7.9 (5.2 to 10.4 ) letters in the treat-and-extend arm, which was non-inferior to + 8.1 (6.5 to 10.6 ) letters in the fixed dosing arm at one year. Fluid free retina was achieved in similar proportions in the two arms (76% in the treat-and-extend arm; 83% in the fixed dosing arm) at one year. In the current report, we evaluate the influence of “traditional” baseline imaging features such as the presence or absence of fluid in different retina compartments as well as novel, volumetric quantitative PCV specific imaging biomarkers on visual and anatomical outcomes.

## Methods

### Study design and population

We analysed clinical and imaging data of participants who were enrolled into a randomized controlled trial in PCV (NCT03117634). Detailed methods and primary outcomes have been published. This study was approved by the SingHealth centralized institutional review board and conducted according to the tenets of the declaration of Helsinki. Written informed consent was provided by all participants prior to study enrolment. No experimental animals were used in the study.

Detailed inclusion criteria and exclusion criteria has been described previously^34^. Briefly, this study compared efficacy of two treatment strategies in eyes with treatment naïve macular-involving PCV. After the initial induction phase with aflibercept monotherapy (week 0,4,8) ,all eyes were randomized to a treat and extend (T&E) retreatment strategy as defined in the protocol and were followed up prospectively for 12 months. All participants were randomized at week 12 after a repeat ICGA and OCT in to either personalised arms or fixed arms at a 3:1 ratio. Participants in the fixed arm went to receive fix doses of 8 weekly aflibercept injections for the remaining duration of the study. In personalised arm participants with a completely regressed polypoidal lesion (PL) on ICGA commenced T&E phase while participant with active PL lesion on ICGA continued 4 weekly aflibercept injections till week 24 and commenced T&E phase thereafter. Retreatments during the T&E phase depended on the protocol specific retreatment criteria.

### Study outcomes and assessment

The outcome measures of interest were: (1) Change in Best Corrected Visual Acuity (BCVA) at month 12 from baseline, which was measured in ETDRS letters and (2) The evidence of disease activity [defined using “traditional” biomarkers such as the presence of new hemorrhage and/or fluid in the subretinal (SRF) and/or intra-retinal space (IRF) on SD-OCT at month 12 ].

Singapore National Eye Centre ocular reading centre (SORC) acted as a centralised reading centre (CRC) for confirmation of the diagnosis of PCV (Everest criteria)^35^ prior to randomization in the treatment trial and for all image analysis and grading for the purposes of this study. The analysis of all quantitative and qualitative features were performed by two independent trained graders who were masked to treatment and BCVA. Disagreements between graders were resolved through open arbitration with supervising specialists*.* All patients underwent colour fundus photo (CFP) , OCT (SD-OCT and SS-OCT), fundus fluorescein angiography (FFA) and indocyanine green angiography (ICGA) . Imaging acquired at baseline, month 3 and month 12 were used in this study.

### Image acquisition

The Colour fundus photo (CFP) were acquired on digital mydriatic retinal camera (TRC-50X/IMAGEnet 2000, Topcon, Tokyo, japan). The SD-OCT raster scans with enhanced depth imaging were acquired on a 30° × 20° (9 × 6 mm) macular region centred on the fovea, in the high speed mode, with 25 B-scans per volume scan. Each B-scan was averaged (ART mode) using 9 frames using spectralis OCT (Heidelberg Engineering, Heidelberg, Germany). A repeat OCT was acquired on a swept source OCT (SS-OCT) platform (Topcon DRI OCT Triton /version 10.17.003.03, Tokyo, japan). The SS-OCT scans were acquired on a 6 × 6 mm scan centred on the fovea with a 1050 nm wavelength scanning laser to capture 100,000A scans per second allowing a rapid and greater penetration of vitreous , retina , choroid and sclera compared to SD-OCT. Dye angiography (FFA and ICGA) were acquired by a confocal scanning laser ophthalmoscope (cSLO) (Spectralis OCT, Heidelberg Engineering, Heidelberg, Germany).

### Image assessment and grading criteria

Sub-retinal blood was defined as the presence of retinal haemorrhage more than 4 disc diameter in-area on the CFP. Area of the Sub-retinal blood was measured using the inbuild measuring tool available in IMAGEnetR4 digital imaging system. (version 4.2.1, Topcon, Tokyo, japan ).

Baseline BNN and PL areas were measured using the free hand drawing tool of the spectralis heidelberg eye explorer (HEYEX) image management platform (version 1.10.4.0) and the region encompassed by the lesion was outlined on selected ICGA frames captured between 5–6 min.

CVH was assessed based on comparison of baseline, mid-phase (5–7 min) and late-phase ICGA (10–15 min) ICGA. We defined CVH as marked if there is presence of patchy hyper fluorescence with blurred margins persisted from the mid to the late phase ICGA, mild if there is fuzziness of choroidal vessels in mid-phase ICGA but no patchy hyper fluorescence in late phase, and absent if none of the above features was present.

All the SD-OCT scans were graded for presence of SRF, IRF and PED. SRF was defined by an area of round or oval hypo-reflectivity with clear borders located within the retinal tissue. IRF was defined as widening of the retinal bands as compared to normal healthy retina. PEDs were defined as focal elevations of the reflective retinal pigment epithelium (RPE) band over an optically clear or moderately reflective space. For this study, a minimum height of 100 µm for PED was used^29^. Quantitative measures were done using the calliper function on HEYEX image management platform (version 1.10.4.0). The quantitative features measured using this calliper function included central retinal thickness (CRT), PED height and choroidal thickness (CT). Central retinal thickness was obtained by automated measurement of the HEYEX software as the distance between the ILM and the outer RPE boundary at BM at the fovea. Maximum PED height (µm) was measured as the vertical measurement between the outer border of the BM and the inner border of the RPE of the highest PED. The CT was measured manually from the outer margin of the RPE to the CSI in the fovea (central 1 mm).

#### Volumetric measurements

**Figure 1:**
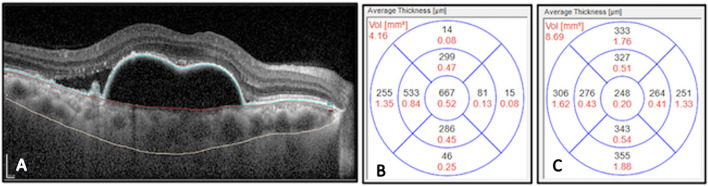
EDI-OCT segmentation of retinal layers and Quantification of PED and choroid thickness and volume. Manual segmentation (**A**) of retinal layers: outer margin of the Retinal Pigment Epithelium (blue line), inner margin of the Bruch’s membrane (red line) and choroid-scleral interface (white line) to generate both volume (mm^3^) within the central 6 mm OCT ETDRS grid and thickness (µm) maps of (**B**) PED volume and (**C**) choroid thickness in treatment naïve eyes with PCV.

The PED volume was derived from RPE-BM volume map generated automatically by the HEYEX software and reflected as PED volume within the central 6 mm ETDRS grid. The software automatically segmented the outer margin of the RPE and inner margin of the BM and computed the volume using the 25 line raster OCT scans. The accuracy of all the automated segmentation was checked by graders who manually adjusted in case of inaccurate placement (Fig. 1).

Macular Choroidal volume (CV) was measured based on a volumetric map between the BM and the choroid scleral interface (CSI). The CSI segmentation line was manually placed (JJ) within the central 6 mm zone, the subsequent volume map was generated automatically and CV computed using the 25 line raster OCT scans. All volumetric measurements were generated at baseline, month 3 and month 12.

#### Choroidal vascular index (CVI)

**Figure 2:**
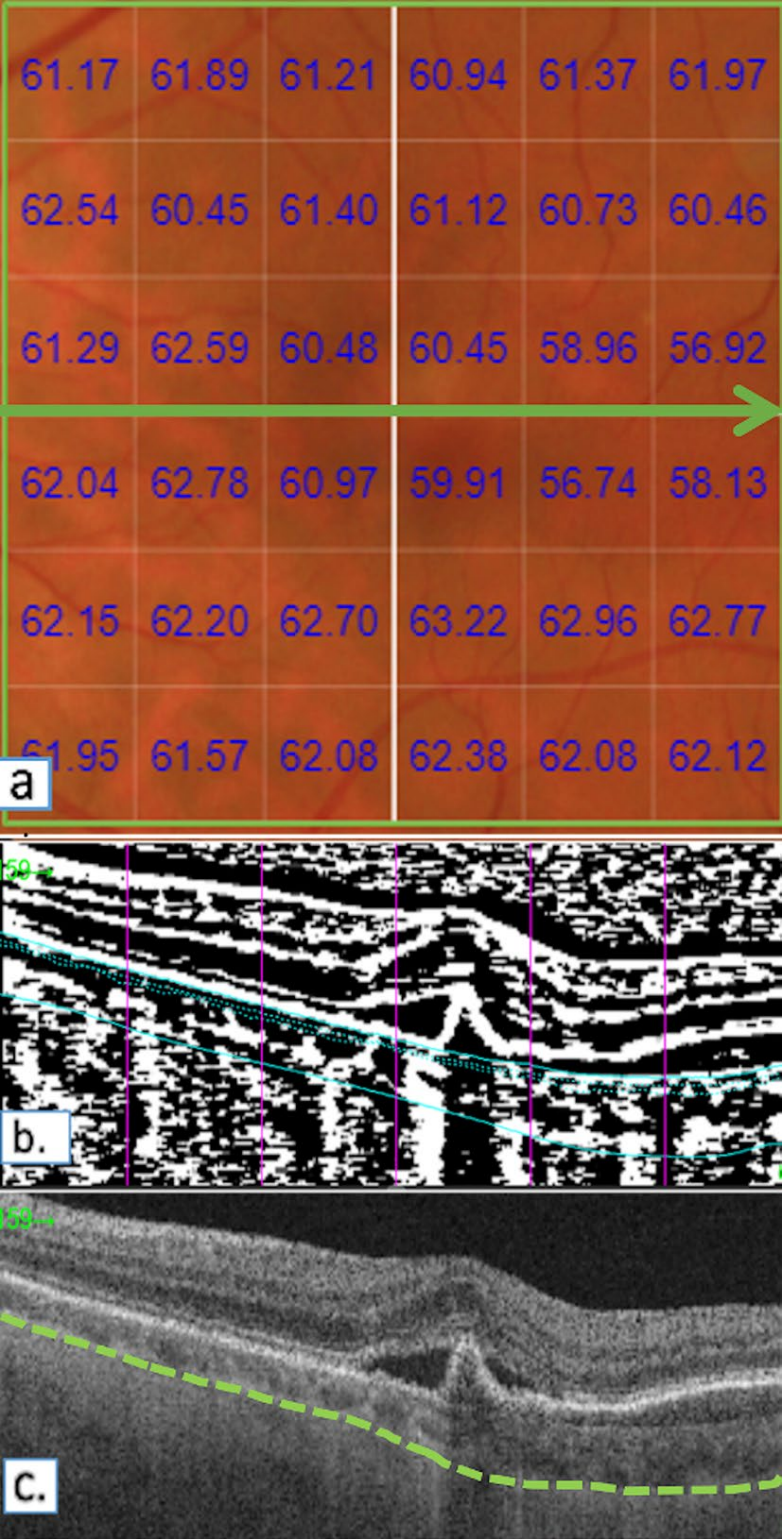
Choroidal vascularity index values generated using structural scans acquired with the swept- source OCTA protocol. (**a**) Automatically generated volumetric CVI values superimposed over the 6 × 6 mm macular area. (**b**) Representative binarized SS-OCT image using Niblack binarization technique at the location of the green arrow. The vertical pink lines indicate the position along the B-scan, in relation to the En-face image. The solid blue lines indicate the inner and outer borders of the choroidal segmentation. i.e. bruch’s membrane and choroid-scleral interface respectively. The dotted blue further segments the ‘inner choroid’. (**c**) Original SS-OCT image at the green arrow. The green line represents the manually marked choroid-scleral interface.

CVI values were generated using structural scans acquired with the swept-source OCTA protocol (Topcon, Japan). Four single B-scan images were captured in same location and were overlapped and further averaging method was applied with consecutive five frames to generate representative B-scan to increase the signal to noise ratio. BM and CSI were automatically delineated with beta segmentation algorithm and manually adjusted by graders if inaccurate. Binarization technique was applied using Niblack method. CVI was expressed as the luminal area/total choroidal area was generated for each B-scan. Volumetric CVI for the 6 × 6 mm scanned area centered over the fovea was calculated as the average CVI of all individual B-scans (Fig. 2).

### Impact of the change from baseline to month 3 in OCT determined macular morphology on month 12 outcomes

Baseline morphological parameters were initially assessed and those found to be significant (*p* < 0.05) were further studied at month 3 to understand the association of change in these factors from baseline to treatment outcomes at month 12. Features that were analysed included baseline to month 3 1) Mean change in CRT (per 100um), 2) Mean change in PED volume (per 100 nl) 3) Mean change in PED height (per 100um) and 4) Mean change in CV (per 100 nl).

### Statistical analysis

We summarized continuous data with means and standard deviations (SD), while categorical variables with counts and percentages. Within group changes from baseline to month 12 were assessed using a paired *t*-test and Mc-Nemar’s Test for continuous and categorical variables, respectively. Comparisons between groups were evaluated using independent T-tests and Chi-Square Tests, respectively for continuous and categorical data.

The BCVA change and disease activity status at month 12 were defined as the two main response variables. Baseline characteristics like patient factors (age , gender), baseline BCVA, ocular factors ( baseline CRT, total lesion area, branching neovascular network (BNN) area , PL area, PED height, PED volume , CT, CV, CVH , CVI) and treatment given (injection number) were analysed against these response variables to establish a correlation. Backward stepwise regression was used to reduce the number of variables and the final model was built on the final variables derived from this stepwise regression. Variables with significance level of *p* < 0.05 were retained for further analysis. Multiple linear regression was used for the final model to describing changes in BCVA and estimated coefficient along with their 95% CI and *p*-values. For the association of disease activity adjusted odds ratios (ORs) with 95% CI and *p*-values were presented for each factor in multiple logistic regression models. A repeat analysis was performed to analyse the relationship between these response variables and month 3 treatment outcomes. Statistical analyses were performed using the Stata statistical software: Release 16 (StataCorp LLC, College Station, TX, USA). Statistical significance was set at less than 0.05.

## Results

Fifty-two eyes from 52 participants were included in the study. The mean age of participants was 69.2 ± 8.6 (range: 49–87) years, and 63.5% (33) were male. Comparison of features between baseline and month 12 are summarized in Table 1. From baseline to month 12, the mean BCVA increased from 61.1 ± 13.2 to 69.6 ± 13.2 ETDRS letters (*p* < 0.01) and the mean CRT reduced from 455.7 ± 182.4 µm to 272.7 ± 86.2 (*p* < 0.01). The proportion of eyes with SRF reduced significantly from 94.2% (49) to 17.3% (9) (*p* < 0.01), while proportion of eyes with IRF reduced from 15.4% (8) at baseline to 11.5% (6) (*p* = 0.6). The proportion of eyes with PED decreased significant from 100 to 80%, *p* < 0.01, the mean maximum height of PED reduced significantly from 381.3 ± 236.3 µm to 206.8 vs ± 146.4 µm (*p* < 0.01) and mean PED volume reduced significantly from 1322 ± 853 nl to 686 ± 593 nl. Choroidal thickness (CT) did not show significant change from baseline to month 12, but choroidal volume (CV) of the whole scan area reduced significantly from 6.7 ± 2.0 mm^3^ to 6.3 ± 2.4 mm^3^ from baseline to month 12 (*p* = 0.02). Similar associations were demonstrated when eyes were stratified according to treatment arms.

**Table 1:** Comparison of features between baseline and month 12.

		Baseline	Month 12	*p*
Age, years	Mean (SD)	69.2 (8.6)	–	–
Gender, female	n (%)	33 (63.5)	–	–
Best corrected visual acuity, letters	Mean (SD)	61.1 (13.2)	69.6 (14.3)	< 0.01
Best corrected visual acuity, letters	Median (IRQ)	64 (53, 72)	73 (61, 80)	< 0.01
central retinal thickness, µm	Mean (SD)	455.7 (182.4)	272.7 (86.2)	< 0.01
central retinal thickness, µm	Median (IRQ)	398 (336, 548)	252 (227, 306)	< 0.01
Presence of SRF	n (%)	49 (94.2)	9 (17.3)	< 0.01
Presence of IRF	n (%)	8 (15.4)	6 (11.5)	0.6
Presence PED	n (%)	52 (100.0)	40 (80.0)	< 0.01
PED height, µm	Mean (SD)	381.3 (236.3)	206.8 (146.4)	< 0.01
PED height, µm	Median (IRQ)	332 (225, 462)	185 (155, 225)	< 0.01
PED volume, nl	Mean (SD)	1322 (853)	686 (593)	< 0.01
PED volume, nl	Median (IRQ)	785 (530, 1585)	540 (405, 808)	< 0.01
Choroidal thickness, µm	Mean (SD)	270.9 (96.4)	257.9 (115.0)	0.53
Choroidal thickness, µm	Median (IRQ)	263 (209, 348)	242 (191, 293)	0.21

*SRF* sub-retinal fluid; *IRF* intra-retinal fluid, *PED* pigment epithelium detachment; *p* value calculated using chi-square test, *t* test, Mc-Nemar’s Test or Wilcoxon rank sum test.

### Associations of baseline parameters with BCVA change at month 12 from baseline (Table 2)

**Table 2:** Linear regression analysis of baseline characteristics against BCVA change at month 12.

Feature	Univariate analysis	*p*	Multivariate analysis	*p*
Age	0.32 (− 0.13 to 0.76)	0.16	0.88 (− 1.51 to 3.27)	0.25
Gender	0.13 (− 0.03 to 0.29)	0.11	0.11 (− 0.06 to 0.26)	0.21
**BCVA (per letter)**	− **0.19 (**− **0.59 to** − **0.21)**	** < 0.01**	− **0.28 (**− **3.38 to** − **1.61)**	**0.02**
**Baseline CRT (per 100um)**	− **0.82 (**− **1.22 to** − **0.42)**	**0.03**	− **0.98 (**− **1.56 to** − **0.40)**	**0.04**
Total lesion area (mm^3^)	− 0.75 (− 1.93 to 0.44)	0.21	− 0.65 (− 1.76 to 0.45)	0.59
BNN area(mm^3^)	− 1.18 (− 2.83 to 0.46)	0.16	− 0.92 (− 2.47 to 0.63)	0.55
PL area (mm^3^)	− 0.67 (− 3.83 to 2.49)	0.67	− 0.90 (− 3.84 to 2.04)	0.51
PED height (per 100um)	− 1.04 (− 3.60 to 1.52)	0.1	− 0.73 (− 2.08 to 0.62)	0.5
**PED volume (nl)**	− **1.02 (**− **1.35 to** − **0.68)**	** < 0.01**	− 0.09 (− 0.19 to 0.10)	0.31
Subfoveal Choroidal Thickness (µm)	− 0.76 (− 2.79 to 1.26)	0.45	− 0.47 (− 30.54 to 29.59)	0.95
Choroidal volume (nl)	− 1.66 (− 3.59 to 0.26)	0.09	0.37 (− 4.96 to 5.69)	0.48
Choroidal vascular hyperpermeability				
Absent	Ref		Ref	
Mild	− 4.14 (− 13.33 to 5.05)	0.37	− 3.51 (− 12.66 to 5.65)	0.45
Marked	− 4.64 (− 14.26 to 4.97)	0.33	− 4.73 (− 14.27 to 4.80)	0.32
Choroidal vascularity Index	− 0.49 (− 4.31 to 3.34)	0.78	2.72 (− 20.51 to 25.96)	0.66
Number of injections received	2.56 (− 2.24 to 7.37)	0.29	2.02 (− 3.42 to 7.45)	0.62

*BNN* branching neovascular network, *PL* polypoidal lesion, *CRT* central retinal thickness, *PED* pigment epithelium detachment, *CVH* choroidal vascular hyper-permeability; CVH defined as marked = presence of patchy hyper fluorescence with blurred margins persisting from the mid to the late phase ICGA, mild = fuzziness of choroidal vessels in mid-phase ICGA but no patchy hyper fluorescence in late phase, absent = none of the above features present; *CV* choroidal volume; *CVI* choroidal vascularity index. Multivariate analysis adjusted for age and sex. Bold font denotes parameters significantly influencing the change in BCVA at month 12 from baseline and are retained in the final multivariable model.

Among the baseline features evaluated, lower baseline BCVA (per letter) (β = -0.19, 95%CI − 0.59 to − 0.21, *p* < 0.01) , lower baseline CRT (per 100um) (β = -0.82, 95%CI − 1.22 to − 0.42), *p* = 0.03) and smaller PED volume (β = -1.02, 95% CI − 1.35 to − 0.68, *p* < 0.01) were significantly associated with better vision at month 12. In the multivariate analysis, baseline BCVA (β = -0.98, 95%CI − 3.38 to − 1.61, *p* = 0.02) and baseline CRT (β = -0.98, 95%CI − 1.56 to − 0.40, *p* = 0.04) remained significantly associated with better vision at month 12. None of the choroidal parameters, including baseline choroidal thickness, choroidal volume, CVH and CVI and change in these had significant associations with BCVA at month 12.

### Associations of baseline parameters with disease activity month12 (Table 3)

**Table 3:** Linear regression analysis of baseline characteristics against disease activity status at month 12.

		Active (n = 13)	Inactive (n = 39)	OR (univariable)	*p*	OR (multi-variable)	*p*
Age	Mean (SD)	68.8 (8.2)	69.3 (8.9)	1.01 (0.94–1.09)	0.84	1.00 (0.80–1.21)	0.95
Gender, male	n (%)	8 (61.5)	25 (64.1)	0.90 (0.25–3.45)	1	0.93 (0.69–1.23)	0.87
Baseline lesion characteristics							
Total lesion area (mm^3^)	Mean (SD)	4.2 (4.1)	4.0 (3.0)	0.98 (0.82–1.23)	0.85	0.90 (0.72–1.16)	0.36
BNN area (mm^3^)	Mean (SD)	3.3 (3.4)	2.9 (1.9)	0.93 (0.72–1.23)	0.57	0.87 (0.63–1.20)	0.34
PL area (mm^3^)	Mean (SD)	1.0 (1.0)	1.2 (1.3)	1.17 (0.70–2.65)	0.62	0.82 (0.45–1.23)	0.58
**Baseline CRT (µm)**	**Mean** **(SD)**	**612.0** **(188.0)**	**366.0** **(129.5)**	**0.59** **(0.32**–**0.88)**	** < 0.01**	**0.82** **(0.69**–**0.90)**	**0.02**
Baseline PED characteristics							
**PED height (µm)**	**Mean** **(SD)**	**542.0** **(298.0)**	**242.0** **(150.0)**	**0.67** **(0.53**–**0.88)**	** < 0.01**	**0.87** **(0.63**–**0.94)**	** < 0.01**
**PED volume (nl)**	**Mean** **(SD)**	**2433** **(1064)**	**799** **(457)**	**0.94** **(0.88**–**0.98)**	** < 0.01**	**0.77** **(0.64**–**0.87)**	**0.02**
Baseline Choroidal characteristics							
Choroidal vascular Hyperpermeability (CVH)							
Absent	n (%)	3 (23.1)	20 (51.2)	Ref		Ref	NA
Mild	n (%)	4 (30.8)	12 (23.1)	1.18 (0.76–2.34)	0.55	1.12 (0.80–2.23)	0.58
** Marked**	**n (%)**	**6** **(46.2)**	**7** **(17.9)**	**1.65** **(1.05**–**2.45)**	**0.03**	**1.69** **(1.05**–**3.43)**	**0.04**
Subfoveal Choroidal Thickness (µm)	Mean (SD)	289.0 (104.0)	258.0 (138.0)	0.83 (0.58–1.16)	0.31	0.80 (0.48–1.29)	0.37
Choroidal Volume (nl)	Mean (SD)	7772 (2044)	6293 (1837)	0.66 (0.44–0.93)	0.02	0.74 (0.32–1.42)	0.22
**Choroidal vascularity Index (CVI)**	**Mean** **(SD)**	**63.0** **(1.4)**	**61.8** **(1.4)**	**0.81** **(0.24**–**0.91)**	**0.02**	**0.78 (0.49**–**0.9)**	**0.04**
Number of injections	Mean (SD)	8 (1.0)	8 (1.0)	0.89 (0.38–1.94)	0.8	0.83 (0.53–1.25)	0.08

*BNN* Branching neovascular network, *PL* polypoidal lesion, *CRT* central retinal thickness, *PED* pigment epithelium detachment, *CVH* choroidal vascular hyper-permeability; CVH defined as marked = presence of patchy hyper fluorescence with blurred margins persisting from the mid to the late phase ICGA, mild = fuzziness of choroidal vessels in mid-phase ICGA but no patchy hyper fluorescence in late phase, absent = none of the above features present, *CV* choroidal volume, *CVI* choroidal vascularity index. Multivariate analysis adjusted for age, sex and total number of injections. Bold fonts denote parameters significantly influencing the disease activity at month 12 from baseline and are retained in the final multivariable model.

**Figure 3:**
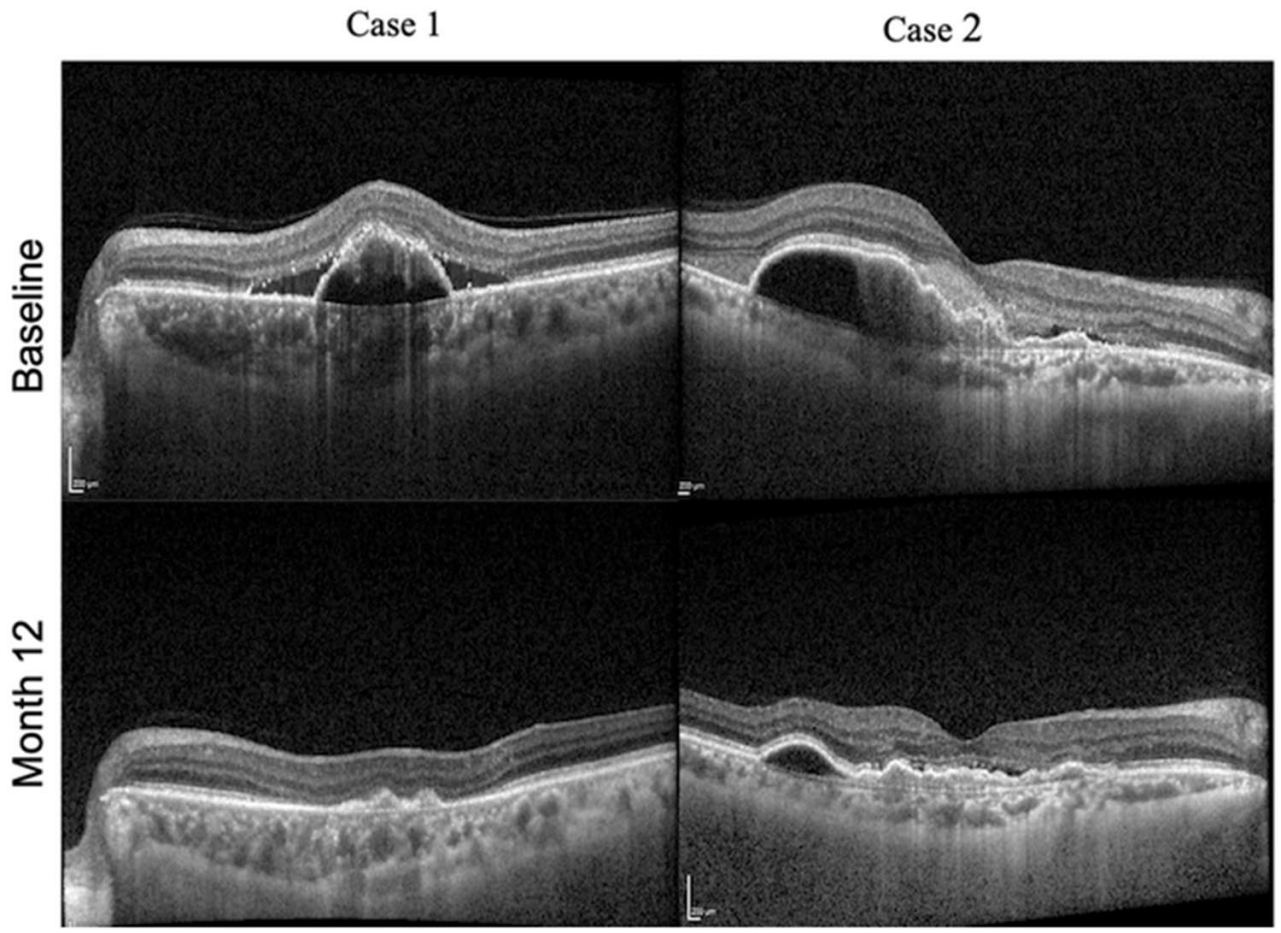
OCT images from 2 eyes showing the change in PED from baseline to month 12. Case 1: Baseline OCT scan showing a large PED with subretinal fluid constituting most of the CRT (367 µm) with PED volume of 0.81 nl and maximum PED height of 338 µm. Month 12 OCT scan showing resolution of PED and subretinal fluid with reduction in CRT (165 ), PED height (67 µm) and PED volume (0.1 nl). Case 2: Baseline OCT scan showing a large sub-foveal PED with SRHM occupying most of the CRT(446 µm) with PED volume of 2.1 nl and maximum PED height of 362 µm) Month 12 OCT scan showing reduction in CRT (220 µm), PED height (171 µm) and PED volume (0.88 nl) along with resolution of SRHM.

At month 12, 25% (13 eyes) had active disease. Associations of baseline characteristics with disease activity at month 12 are summarized in Table 3. Compared to eyes with active disease, eyes with inactive disease at month 12 had lower baseline CRT (366.0 ± 129.5 vs 612.0 ± 188.0 , *p* < 0.001), lower baseline PED height (242.0 ± 150.0 µm vs 542.0 ± 298.0 µm, *p* < 0.01), lower baseline PED volume (0.6 ± 0.3 mm^3^ vs 2.2 ± 1.3 mm^3^ vs, *p* < 0.01), lower proportion with marked CVH (17.9% vs 46.2%, *p* = 0.02) and lower mean CVI (61.8 ± 1.4 vs 63.0 ± 1.4, *p* < 0.02) (Fig. 3). These changes remained significant in the multivariate model.

### Associations of OCT macular morphology change from baseline to M3 with outcomes at month 12 (Table 4)

**Table 4:** Association of treatment outcomes at month 3 with outcomes at month 12.

Response feature	Univariate beta	*p*	Multivariate beta	*p*
**Association with gain in BCVA in letters**				
Baseline to month 3 mean change in CRT (per 100 nm)	**1.67** **(0.45 to 2.90)**	**0.02**	**0.51** **(0.12 to 2.18)**	**0.03**
Baseline to month 3 mean change in PED volume (per 100 nl)	**0.93** **(0.20 to 1.66)**	**0.01**	**1.04** **(0.07 to 2.01)**	**0.03**
Baseline to month 3 mean change in PED height (per 100um)	0.01 (− 0.01 to 0.03)	0.25	− 0.00 (− 0.03 to 0.02)	0.78
Baseline to month 3 mean change in CV (per 100 nl)	− 0.01 (− 1.28 to 1.25)	0.98	0.22 (− 0.96 to 1.40)	0.71
**Association with inactive disease**				
Baseline to month 3 mean change in CRT (per 100 nm)	**1.72** **(1.16 to 2.80)**	**0.01**	**1.32** **(1.03 to 2.82)**	**0.03**
Baseline to month 3 mean change in PED volume (per 100 nl)	**1.42** **(1.18 to 1.79)**	**0.01**	**1.39** **(1.11 to 1.82)**	**0.01**
Baseline to month 3 mean change in PED height (per 100um)	0.91 (0.49 to 1.15)	0.62	1.00 (0.44 to 1.30)	0.98
Baseline to month 3 mean change in CV(per 100 nl)	1.42 (1.05 to 2.03)	0.03	0.83 (0.44 to 1.48)	0.56

*CRT* central retinal thickness, *PED* pigment epithelium detachment, *CV* choroidal volume; Multivariate analysis adjusted for age, sex and number of injections. Bold fonts denotes parameters significantly influencing the gain in BCVA and disease activity at month 12 from baseline and are retained in the final multivariable model.

In addition to baseline features, we also evaluated whether OCT morphological changes from baseline to month 3 was associated with visual and anatomical outcome at month 12. In the multivariate analysis, a larger decrease in CRT and a larger reduction in PED volume at month 3 from baseline were associated with greater BCVA gain at month 12 (CRT β = 0.51 per 100 nm, 95%CI 0.12 to 2.18, *p* = 0.03; PED volume β = 1.04 per 100 nl, 95%CI 0.07 to 2.01, *p* = 0.03) and with inactive disease at month 12(CRT β = 1.32 per 100 nm, 95% CI 1.03 to 2.82, *p* = 0.03; PED volume β = 1.39 per 100 nl, 95%CI 1.11 to 1.82, *p* = 0.01).

## Discussion

In this prospective clinical trial of aflibercept where participants were randomized to either fixed dosing or personalized treat and extend regimen, we identified that the baseline PED volume, PED height, CVH and CVI were significantly associated with disease activity at month 12.

Traditional quantitative metrices that are measured on SD-OCT pertaining to PEDs are its height and width although it is a three-dimensional focal elevation of the reflective RPE band over an optically clear or moderately reflective space and is one of the most distinct features of PCV^29^. With recent advances in segmentation algorithms, volumetric analysis of retinal pathological features such as IRF, SRF and PED have been suggested as useful biomarkers for predicting treatment response^36^. We measured the PED volume and included this parameter amongst the other traditionally acquired explanatory variables in the multiple regression model of disease activity which was the dependent variable. PED volume was retained in the final model, indicating that it contributes to explaining more of the variance in disease activity than PED height alone. The more accurate assessment of the PED as three-dimensional structure and the detection that a greater reduction in the PED volume was associated with better outcomes is therefore of clinical value and importance (Fig. 3).

Another key feature of PCV is the choroidal architecture. Dilated choroidal vessels and choroidal hyperpermeability have been hypothesized etiological processes that lead to PCV^37,38^. A novel feature that we evaluated was the volumetric CVI. While some prior studies have evaluated CVI based on a single subfoveal B-scan OCT, the current study evaluated volumetric CVI across the 6 × 6 mm macular area^39^. Our data suggest that a higher volumetric CVI is associated with active disease at month 12. The second choroidal feature we examined was CVH. We found that marked CVH was significantly associated with disease activity however neither an adverse nor beneficial association between CVH and visual outcome was noted at month 12. This is consistent with the equivocal results from studies have assessed treatment outcomes and choroidal characteristics such as sub-foveal choroidal thickness and CVH^40–44^. A recent study reported that the presence of CVH favorably influenced visual outcome with lower number of anti VEGF treatments combined with PDT^14^. However other studies have shown that the presence of CVH was associated with poor visual and anatomical outcomes after treatment with anti VEGF^31,44^.

CVI and CVH may reflect the structural and functional alterations, respectively, in eyes with PCV and may be surrogate markers indicative of choroidal congestion. More severe alteration in these parameters as a cause or consequence of disease indicate a more extensive choroidal involvement with pathological alterations of choroidal vascular architecture and may hence be associated with poorer outcomes with treatment.

In addition to morphology at baseline, we also evaluated if the treatment response on completion of the loading phase influenced the visual and disease activity outcome at month 12. It was notable that a larger reduction in CRT and PED volume from baseline to month 3 was associated with better visual outcome and disease inactivity at month 12. Therefore, in addition to widely accepted markers of activity like SRF and IRF in typical nAMD, we postulate that PED volume is an important imaging biomarker that may help in the risk stratification in PCV. This is biologically plausible as sub-RPE fluid accumulation is a reflection of active polypoidal lesion(s) and/or the associated type-1 neovascular network.

The strengths of this study are its prospective collection of robust data, with morphology graded in the reading center by trained graders who were masked to clinical information and who used a pre-specified grading protocol. All the volumetric readouts were meticulously checked to ensure that SD-OCT segmentation was accurate as distortion of the RPE is common in eyes with PCV. Our study has a number of limitations. Our sample size was a moderate-sized cohort. The grading of CVH is subjective and can be affected by imaging technique. PED volume and CVI measurements were performed using non-commercially available software therefor have limited generalizability. One of the limitations of CVI measurement currently is the accuracy in defining the CSI. The choroidal segmentation can be challenging in eyes with thick choroids, especially in eyes with overlying retinal pathology which results in masking of CSI. In addition, there is currently no ideal binarization algorithm to determine the CVI especially in cases where the visualization of the choroid is affected by the overlying pathology. In this study, eyes with poor quality scans were excluded from CVI analysis. Each b scan included was assessed for segmentation accuracy and corrected manually if necessary.

In conclusion, the current analysis identified PCV-specific features that predict treatment outcomes and likely to be useful in guiding retreatment decisions. This preliminary data demonstrates the potential for PED-related volumetric parameters as an additional marker of disease activity however further studies are needed to establish its usefulness in retreatment decisions. With the increasing popularity of anti VEGF monotherapy in the treatment for PCV, these novel findings may improve treatment success in personalized retreatment regimens.
